# Lead-free perovskite solar cells using Sb and Bi-based A_3_B_2_X_9_ and A_3_BX_6_ crystals with normal and inverse cell structures

**DOI:** 10.1186/s40580-017-0120-3

**Published:** 2017-09-22

**Authors:** Ajay Kumar Baranwal, Hideaki Masutani, Hidetaka Sugita, Hiroyuki Kanda, Shusaku Kanaya, Naoyuki Shibayama, Yoshitaka Sanehira, Masashi Ikegami, Youhei Numata, Kouji Yamada, Tsutomu Miyasaka, Tomokazu Umeyama, Hiroshi Imahori, Seigo Ito

**Affiliations:** 1 Department of Materials and Synchrotron Radiation Engineering, Graduate School of Engineering, University of Hyogo, 2167 Shosha, Himeji, Hyogo 671-2280 Japan; 2 Graduate School of Engineering, Toin University of Yokohama, Yokohama, Kanagawa 225-8503 Japan; 3 Department of Applied Molecular Chemistry, College of Industrial Technology, Nihon University, 1-2-1, Izumi-Chou, Narashino-Shi, Chiba, 275-8575 Japan; 4 Department of Molecular Engineering, Graduate School of Engineering and Institute for Integrated Cell Materials Sciences (WPI-iCeMS), Kyoto University, Nishikyo-Ku, Kyoto, 615-8510 Japan

**Keywords:** Lead-free perovskite, Solar cells, Antimony, Bismuth

## Abstract

Research of CH_3_NH_3_PbI_3_ perovskite solar cells had significant attention as the candidate of new future energy. Due to the toxicity, however, lead (Pb) free photon harvesting layer should be discovered to replace the present CH_3_NH_3_PbI_3_ perovskite. In place of lead, we have tried antimony (Sb) and bismuth (Bi) with organic and metal monovalent cations (CH_3_NH_3_^+^, Ag^+^ and Cu^+^). Therefore, in this work, lead-free photo-absorber layers of (CH_3_NH_3_)_3_Bi_2_I_9_, (CH_3_NH_3_)_3_Sb_2_I_9_, (CH_3_NH_3_)_3_SbBiI_9_, Ag_3_BiI_6_, Ag_3_BiI_3_(SCN)_3_ and Cu_3_BiI_6_ were processed by solution deposition way to be solar cells. About the structure of solar cells, we have compared the normal (n-i-p: TiO_2_-perovskite-spiro OMeTAD) and inverted (p-i-n: NiO-perovskite-PCBM) structures. The normal (n-i-p)-structured solar cells performed better conversion efficiencies, basically. But, these environmental friendly photon absorber layers showed the uneven surface morphology with a particular grow pattern depend on the substrate (TiO_2_ or NiO). We have considered that the unevenness of surface morphology can deteriorate the photovoltaic performance and can hinder future prospect of these lead-free photon harvesting layers. However, we found new interesting finding about the progress of devices by the interface of NiO/Sb^3+^ and TiO_2_/Cu_3_BiI_6_, which should be addressed in the future study.

## Introduction

Recent advancements of organic–inorganic perovskite (CH_3_NH_3_PbI_3_) thin-film solar cells, which can be fabricated by economical-promising solution process, have marked significant achievements of photoconversion efficiency (PCE) from the initial efficiency of 3.8% to over 22% [1–3]. The PCE progress has been realized due to the excellent ability of CH_3_NH_3_PbI_3_ about light harvesting, charge separation and charge transportation. These excellent properties also realize to fabricate perovskite solar cells in different structural designs as normal (n-i-p) mesoscopic and inverted (p-i-n) planar configurations. However, the working instability had been a profound problem [4–7]. Moreover, the toxicity of lead (Pb) shows big impact on the environment and human being, and then, the quest for such lead-free thin film solar cell has been highly demanded [8, 9]. The first lead-free perovskite solar cells were initially fabricated with the substitution of lead to tin (Sn) for the mesoscopic structure solar cells as <FTO glass/cp-TiO_2_/CH_3_NH_3_SnI_3_/Spiro-OMeTAD/Au> [10, 11]. A moderate PCE of 6% was reported, but the rapid oxidation of Sn^2+^ to be Sn^4+^ at the ambient condition arised concern. Afterward, however, it was found that tin cation shows higher toxicity than lead [12]. Hence, another elements have been considered for the further ongoing lead-free solar cells.

In order to substitute the Pb, another potential, germanium (Ge^2+^) cation was implemented into the organo-metal-halide crystal as a harvesting layer, which has the same oxidation state and lower electronegativity and resulted in 0.2% PCE [13]. As another divalent, earth abundant and non-toxic element, copper cation was utilized in mesoscopic perovskite solar cell structure with (CH_3_NH_3_)_2_CuCl_x_Br_y_ as a photon harvesting layer [14]. The low PCE of 0.017% was observed due to the low absorption coefficients, the high effective mass of holes and the low intrinsic conductivity of the employed perovskite layer.

The other viable opinion towards lead cation (Pb^2+^) substitution is adaptation of heterovalent (not divalent) cation into the organic-metal-halide crystals. These substitutions have to follow charge neutrality and can alter perovskite structure (ABX_3_) to another crystal (A: organic ion; B: metal ion; X: halide ion). Recently, trivalent cations of antimony (Sb) and bismuth (Bi) based organic-metal-halide crystals (A_3_B_2_X_9_ or A_3_BX_6_) got significant interest as a photon harvesting layer due to their environmentally friendly nature and available inactive outer shell s orbital [15, 16]. Öz et al. proposed (CH_3_NH_3_)_3_Bi_2_I_9_ based inverted solar cells as <ITO/PEDOT:PSS/(CH_3_NH_3_)_3_Bi_2_I_9_/PCBM/Ca/Al. However, the energy mismatch limited the charge extraction, resulting in the low PCE to 0.07%. Hebig et al. proposed <ITO/PEDOT:PSS/(CH_3_NH_3_)_3_Sb_2_I_9_)/PCBM/ZnO/Al> structures and reported 0.49% PCE [15, 16]. The trivalent bismuth (Bi^3+^) has utilized for the fabrication of (CH_3_NH_3_)_3_Bi_2_I_9_ based organic-metal-halide layer, which showed optimal absorption coefficient [17, 18]. This Bi-based organic-metal-halide solar cells were reported 0.33% of the low PCE, which was limited by its high exciton binding energy of 300 meV. It was founded that the morphology of (CH_3_NH_3_)_3_Bi_2_I_9_ was changed by the selection of electron transporting layers (ETL), and that the small amount addition of NMP (*N*-methyl-2-pyrrolidone) solvent could also attain to grow uniform surface morphology [18, 19].

In order to improve the Bi-based A_3_BX_6_-structured solar cells, recently, Ag and Cs were composed as A-site monovalent cations to be Cs_2_AgBiX_6_ structure, due to better optical and electrical properties and its ambient stability [20–23]. The substitution of methylammonium ion with silver can extend the dimension of all inorganic active material maintaining its 3d structure, which can be more suitable for photo harvesting due to its uniform nature. Ag found application in various structural compounds including AgBiI_4_ and (CH_3_NH_3_)_2_AgBiBr_6_ although, only optical properties are established and the PCE is yet to be demonstrated [24, 25]. Moreover, the Chemical materials Evaluation and REserarch BAse (CEREBA) reported the incorporation of the silver based A-site cation by replacing the methylammonium (CH_3_NH_3_^+^) ion preserving the Bi and iodine (Ag_3_BiI_6_) and reported 4.3% PCE [23]. Hence, it can be assumed that the Ag–Bi based photo absorber can perform the lead free organo-metal-halide solar cells with better PCE.

**Fig. 1:**
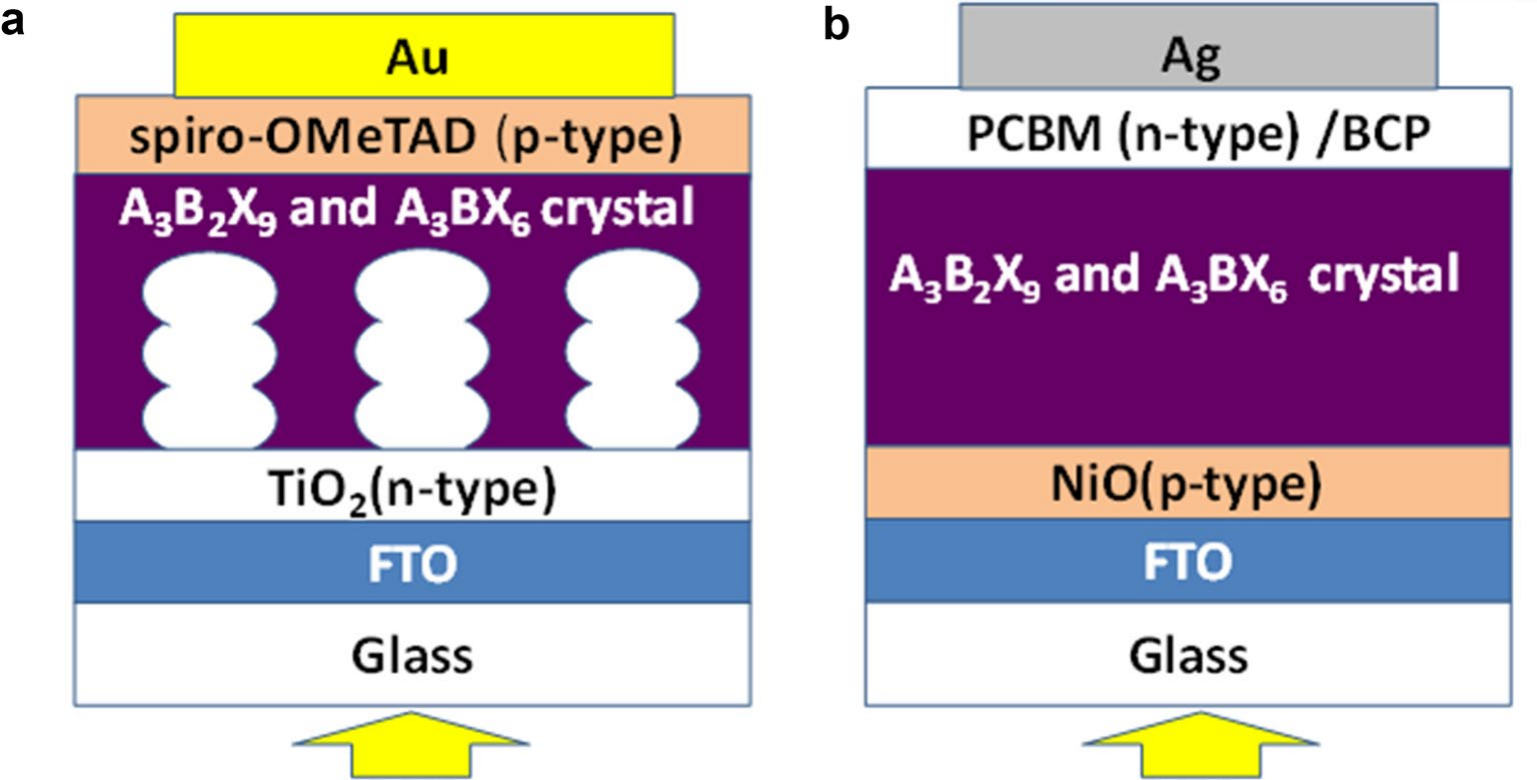
Structures of fabricated solution processed lead-free A_3_B_2_X_9_ and A_3_BX_6_ crystal (A: monovalent cation; B: trivalent cation; X: halogen anion) solar cells: normal mesoscopic (n-i-p) (**a**) and inverted planar (p-i-n) (**b**) structures

In order to improve the photovoltaics of lead free A_3_B_2_X_9_ and A_3_BX_6_ solar cells, the further morphological and structural study of Sb and Bi-based metal-halide layers and the effect on PCE should be studied. In this study, we have tried A_3_B_2_X_9_ and A_3_BX_6_-structured crystals for the photo absorber layers of solar cells with several combinations of A site cations (CH_3_NH_3_^+^, Ag^+^ and Cu^**+**^) and B site cations (Bi^**3+**^ and Sb^3+^), which were (CH_3_NH_3_)_3_Bi_2_I_9_, (CH_3_NH_3_)_3_Sb_2_I_9_, (CH_3_NH_3_)_3_SbBiI_9_, Ag_3_BiI_6_, Ag_3_BiI_3_(SCN)_3_ and Cu_3_BiI_6_. The crystal configurations of A_3_B_2_X_9_ and A_3_BX_6_ were tried to be controlled by the amount of elements in the solutions of source materials. These crystal layers were implemented in normal (n-i-p) and inverted (p-i-n) solar cell architectures (Fig. 1): the normal mesoscopic solar cell <FTO glass/cp TiO_2_/mp TiO_2_/(Pb-free A_3_B_2_X_9_ and A_3_BX_6_ layer)/spiro-OMeTAD/Au> and the inverted structure <FTO glass/NiO layer/(Pb-free A_3_B_2_X_9_ and A_3_BX_6_ layer)/PCBM/BCP/Ag>. It was confirmed that the adjacent substrate to A_3_B_2_X_9_ crystal (TiO_2_ electron transporting layer (ETL) and NiO hole transporting layer (HTL)) affected on the lead-free A_3_B_2_X_9_ and A_3_BX_6_ morphology and its effect on PCE are studied.

## Results and discussion

### A_3_B_2_X_9_ crystals with methyl ammonium cation for the A site

**Fig. 2:**
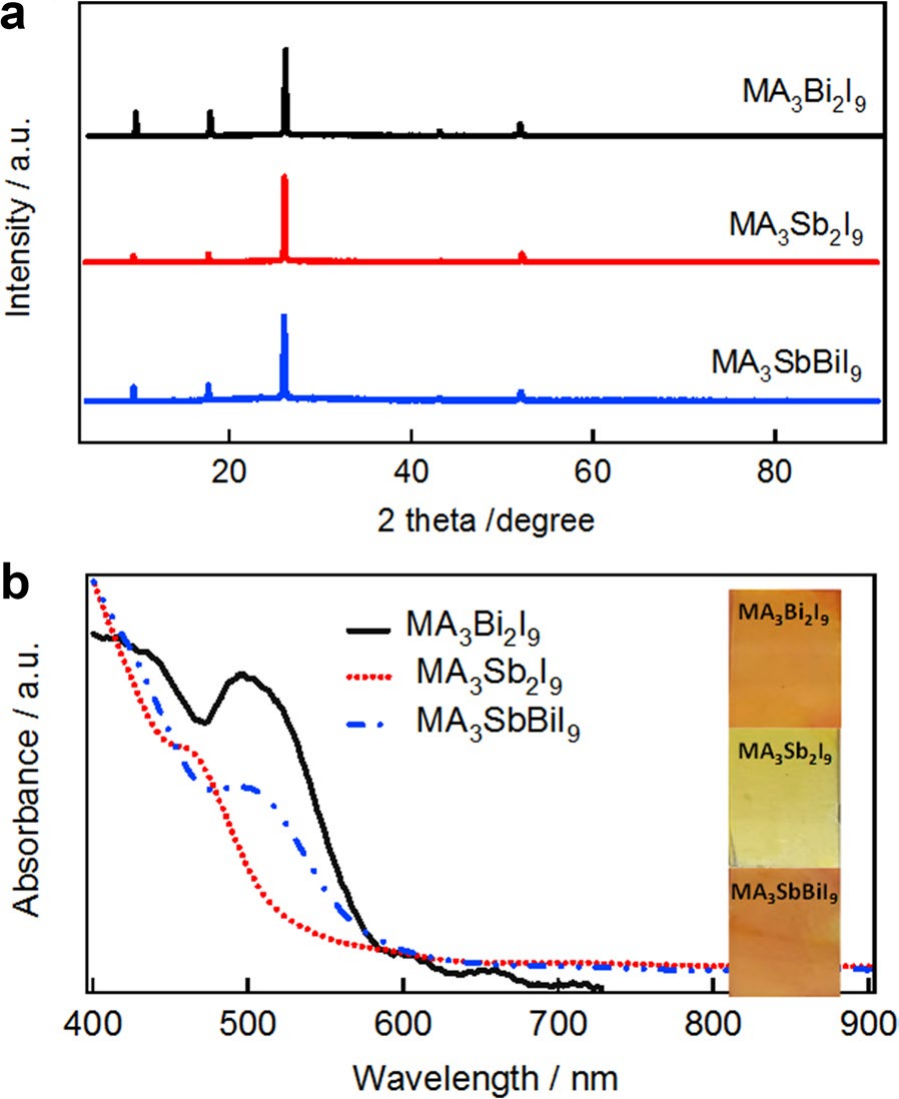
XRD patterns (**a**) and UV–vis absorption spectra (**b**) of grown solution processed (CH_3_NH_3_)_3_Bi_2_I_9_, (CH_3_NH_3_)_3_Sb_2_I_9_ and (CH_3_NH_3_)_3_SbBiI_9_ films on a glass substrate. The insets were the pictures of (CH_3_NH_3_)_3_Bi_2_I_9_, (CH_3_NH_3_)_3_Sb_2_I_9_ and (CH_3_NH_3_)_3_SbBiI_9_ films

The XRD patterns of (CH_3_NH_3_)_3_Bi_2_I_9_, (CH_3_NH_3_)_3_Sb_2_I_9_, and (CH_3_NH_3_)_3_SbBiI_9_ thin films are similar and possesses the strong preferential growth in c axis direction (Fig. 2a). The observed XRD peaks of thin (CH_3_NH_3_)_3_Bi_2_I_9_ film at 8.16, 16.34 and 24.62 are well matched to the literature and correspond to the indexed planes (002), (004) and (006) respectively in a P6_3_/mmc hexagonal space group where preferential growth direction is in c axis direction [15]. The observed peak of (CH_3_NH_3_)_3_Sb_2_I_9_ film at 8.22, 16.54, 24.88 and 50.98 could be indexed by planes (001), (002), (003) and (402) respectively in P6_3_/mmc hexagonal space group and possesses the strong preferential growth in c axis direction similar to the (CH_3_NH_3_)_3_Bi_2_I_9_ film [16]. The XRD pattern of (CH_3_NH_3_)_3_SbBiI_9_ film containing Bi and Sb also shows preference to have the same c axis orientation growth. Due to the multi cation at B site in the crystal, we could not define the exact element at each position. Hence, we should not put the exact index at the peaks. The details analysis is ongoing for the next publication as the future works.

Figure 2b shows UV–vis spectra and photographs of (CH_3_NH_3_)_3_Bi_2_I_9_, (CH_3_NH_3_)_3_Sb_2_I_9_, and (CH_3_NH_3_)_3_SbBiI_9_ thin films. The (CH_3_NH_3_)_3_Bi_2_I_9_ film absorbs with the peak appearing around 500 nm. On the other hand, The UV–vis spectrum of (CH_3_NH_3_)_3_Sb_2_I_9_ shows a shoulder peak around 460 nm and no clear peak. The casted (CH_3_NH_3_)_3_SbBiI_9_ film shows higher absorption around 500 nm than (CH_3_NH_3_)_3_Bi_2_I_9_ and (CH_3_NH_3_)_3_Sb_2_I_9_ films. The exact absorption coefficients were not shown in the figure due to the inhomogeneous structures of films, which will be shown later.

**Fig. 3:**
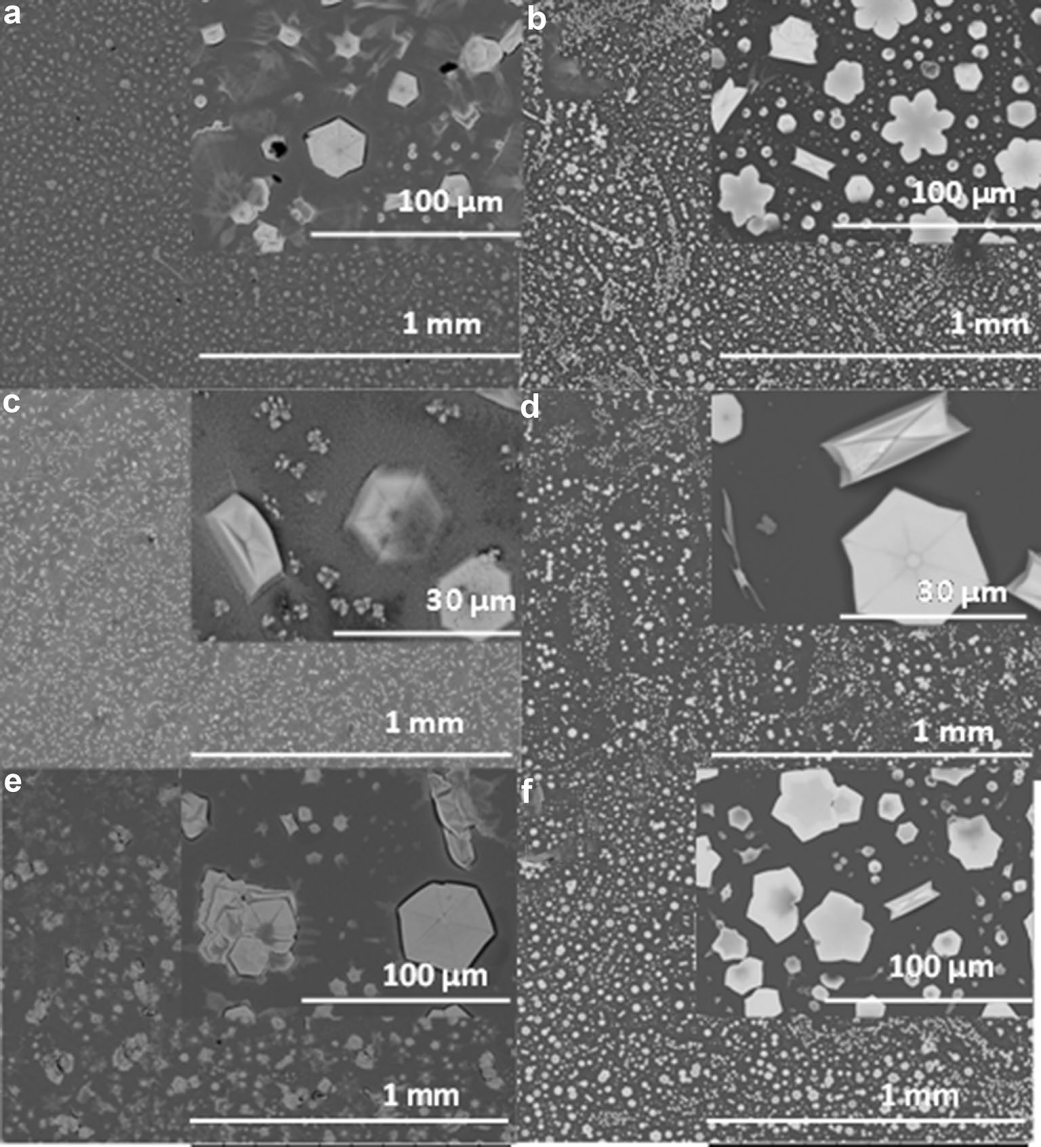
SEM images of A_3_B_2_X_9_ films [(CH_3_NH_3_)_3_Bi_2_I_9_: (**a**, **b**); (CH_3_NH_3_)_3_Sb_2_I_9_: (**c**, **d**); (CH_3_NH_3_)_3_SbBiI_9_: (**e**, **f**)] grown on different substrates [TiO_2_: (**a**, **c**, **e**); NiO: (**b**, **d**, **f**)]

The (CH_3_NH_3_)_3_Bi_2_I_9_, (CH_3_NH_3_)_3_Sb_2_I_9_, and (CH_3_NH_3_)_3_SbBiI_9_ layers processed on different substrates (TiO_2_ or NiO) were observed with scanning electron microscopy (SEM) images (Fig. 3). Due to the crystal structures with hexagonal space group detected by XRD (Fig. 2a), the morphologies became also hexagonal shapes, basically. The (CH_3_NH_3_)_3_Bi_2_I_9_ film growth morphology on TiO_2_ layer is hexagonal (Fig. 3a), but were irregular hexagon and star shape on NiO film (Fig. 3b). Apparently, this observed variation of morphology pattern is attributed to the interface between metal oxides (TiO_2_ and/or NiO) and A_3_B_2_X_9_ organic-metal-halide crystals. On the other hand, the (CH_3_NH_3_)_3_Sb_2_I_9_ (Fig. 3c, d) crystals were hexagonal simply, irrespective of substrate (TiO_2_ and/or NiO). Additionally, on the NiO substrate, the appearing irregular hexagon shows its polycrystalline nature. About the (CH_3_NH_3_)_3_SbBiI_9_ film (Fig. 3e, f), these observed SEM images provide the information of the surface coverage, suggesting polycrystalline growth of hexagonal and irregular hexagonal in nature. Essentially, the inhomogeneity is involved in the growth of film, and the homogeneous surface coverage is difficult. This characteristics may be attributed to the poor PCE and with polycrystalline growth nature [26].

**Fig. 4:**
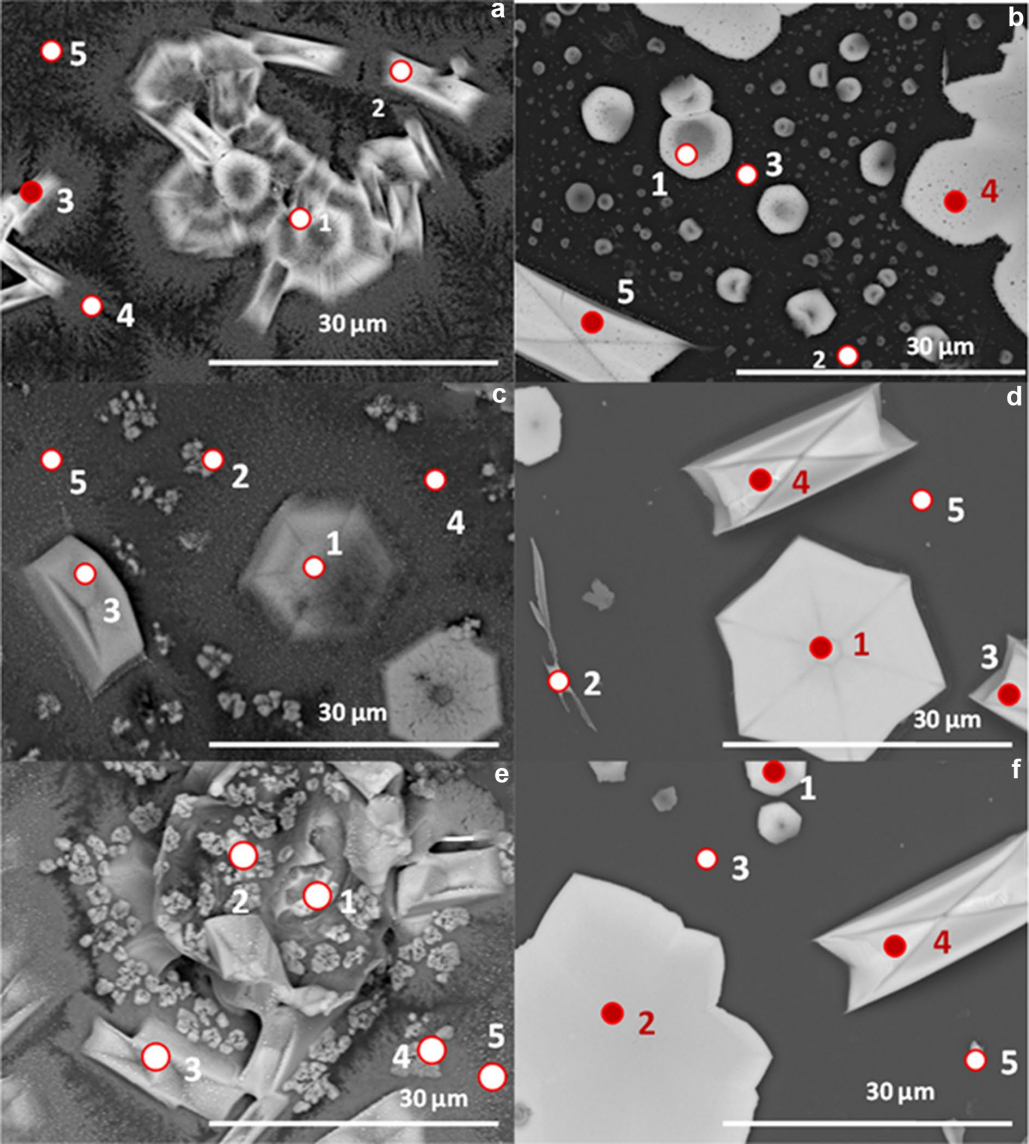
SEM images observed for elemental analysis of A_3_B_2_X_9_ films [(CH_3_NH_3_)_3_Bi_2_I_9_: (**a**, **b**); (CH_3_NH_3_)_3_Sb_2_I_9_: (**c**, **d**); (CH_3_NH_3_)_3_SbBiI_9_: (**e**, **f**)] grown on different substrates [TiO_2_: (**a**, **c**, **e**); NiO: (**b**, **d**, **f**)]. The marked spots indicate the positions of elemental analysis, which are related to the results shown in Tables 1, 2, 3, 4, 5, and 6

Figure 4 shows the SEM images of (CH_3_NH_3_)_3_Bi_2_I_9_, (CH_3_NH_3_)_3_Sb_2_I_9_, and (CH_3_NH_3_)_3_SbBiI_9_ crystals casted on TiO_2_ and NiO substrates for the elemental distribution by EDX analysis. In order to reduce the electron charge accumulation on the surface during the EDX measurements, the gold (Au) thin layer was deposited by sputtering system beforehand. Hence the component contribution from Au was observed. The sum of all elemental contribution was set to be 100. The points of elemental analysis were chosen at various particular spot locations and are numbered in Fig. 4. The results of elemental analysis are summarized in Tables 1, 2, 3, 4, 5, 6, which are projected by the numbers in Fig. 4. About (CH_3_NH_3_)_3_Bi_2_I_9_ film elemental analysis on TiO_2_ substrates (Fig. 4a, Table 1), the small amounts of Bi and I out of grain were observed (points 4 and 5). The points on grain (point 1, 2, and 3) show the large amount of Bi and I than those out of grain (points 4 and 5). Hence, It can be confirmed that there was also the thin layer of (CH_3_NH_3_)_3_SbBiI_9_ at the points out of the large grain. The ratios of I/Bi at the points 1, 2, 3, 4, and 5 in Fig. 4a were 4.21, 3.91, 3.76, 4.08 and 4.25, respectively. Thinking about the I/Bi stoichiometry ratio (= 4.5) in the (CH_3_NH_3_)_3_Bi_2_I_9_ crystal, the results of EDX show the reduction of iodide element in the material. Although Bi was distributed on all points of the TiO_2_ surface (Table 1), there are missing points of Bi on NiO layer (at the points 2 and 3 in Table 2). The points 2 and 3 out of grain on NiO may possess only CH_3_NH_3_I. The ratios of I/Bi at the points 1, 4, and 5 in Fig. 4b and Table 2 are 4.92, 4.13, and 4.02, respectively, which are larger than those of grains in Fig. 4a and Table 1. Possibly, the solution of (CH_3_NH_3_)_3_Bi_2_I_9_ was repelled from the surface of NiO and crystallized on NiO close to the stoichiometry ratio. About (CH_3_NH_3_)_3_Sb_2_I_9_, it was surprising that there are missing points of Sb not only on NiO, but also on TiO_2_ (Fig. 4c, d, Tables 3, 4). Therefore, the anchoring strength of Sb on to the oxides (TiO_2_ or NiO) would be weaker than that of Bi. The elemental ratios (I/Sb) of crystals were 6.17 and 4.79 on TiO_2_ (at the points 1 and 3 in Fig. 4c and Table 3) and 3.98, 3.65 and 4.03 on NiO (at the points 1, 3 and 4 in Fig. 4d and Table 4), respectively. The reason was not clear, but the elemental ratios (I/Sb) of crystals were higher on TiO_2_ than NiO. About (CH_3_NH_3_)_3_SbBiI_9_ (Fig. 4e, f, Tables 5, 6), Bi was not observed out of the crystal as (CH_3_NH_3_)_3_Sb_2_I_9_ (Fig. 4c, d, Tables 3, 4), which was predicted by the weak anchoring strength between Sb and metal oxides as above. Although the material ratio in the solution of (CH_3_NH_3_)_3_SbBiI_9_ was in the stoichiometry, the elemental ratio of Sb/Bi was changed very much to the crystals, which would be due to the segregation to (CH_3_NH_3_)_3_SbI_9_ and (CH_3_NH_3_)_3_BiI_9_. The elemental ratios (I/(Sb + Bi)) of (CH_3_NH_3_)_3_SbBiI_9_ crystals were 4.11, 3.92, and 3.95 on TiO_2_ (at points 1, 2, and 3 in Fig. 4e and Table 5) and 4.92, 7186, 3.92, and 4.52 on NiO (at points 1, 2, 4 and 5 in Fig. 4f and Table 6), respectively. The large variation of elemental ratio [I/(Sb + Bi)] would be attributed to the crystal segregations. Anyway, the SEM–EDX analysis suggests the nonuniformity of the crystals of (CH_3_NH_3_)_3_Bi_2_I_9_, (CH_3_NH_3_)_3_Sb_2_I_9_, and (CH_3_NH_3_)_3_SbBiI_9_. These all results are in contrast to the lab scale established CH_3_NH_3_PbI_3_ perovskite-based solar cells where high efficiencies are achieved with uniform and pin hole free surface morphology [27, 28].

**Table 1:** Elemental distribution (atomic %) of the individual elements observed on particular spot on (CH_3_NH_3_)_3_Bi_2_I_9_/TiO_2_ film shown in Fig. 4a

Spot	Bismuth	Iodine	Titanium	Gold
Point 1	10.00	42.10	45.55	2.72
Point 2	12.87	50.35	34.17	2.59
Point 3	17.25	64.87	14.49	3.11
Point 4	6.32	25.79	64.17	3.29
Point 5	2.79	11.86	80.89	4.02

**Table 2:** Elemental distribution (atomic %) of the individual elements observed on a particular spot on (CH_3_NH_3_)_3_Bi_2_I_9_/NiO film shown in Fig. 4b

Spot	Bismuth	Iodine	Nickel	Gold
Point 1	13.92	68.52	10.88	6.95
Point 2	0.00	11.14	46.54	42.31
Point 3	0.00	12.25	45.62	42.12
Point 4	18.07	74.56	2.33	4.64
Point 5	19.01	76.55	0.92	3.29

**Table 3:** Elemental distribution (atomic %) of the individual elements observed on particular spot on (CH_3_NH_3_)_3_Sb_2_I_9_/TiO_2_ film shown in Fig. 4c

Spot	**Antimony**	**Iodine**	**Titanium**	**Gold**
Point 1	7.71	47.59	38.97	5.71
Point 2	0.00	26.17	66.96	6.86
Point 3	13.06	62.55	22.25	2.12
Point 4	0.00	15.55	78.85	5.37
Point 5	0.00	8.95	89.17	1.66

**Table 4:** Elemental distribution (atomic %) of the individual elements observed on a particular spot on (CH_3_NH_3_)_3_Sb_2_I_9_/NiO substrate shown in Fig. 4d

Spot	Antimony	Iodine	Nickel	Gold
Point 1	19.54	77.83	0.26	2.35
Point 2	0.00	6.83	43.44	49.72
Point 3	20.26	73.91	0.71	5.10
Point 4	19.64	79.22	0.20	0.93
Point 5	0.00	3.84	44.04	52.11

**Table 5:** Elemental distribution (atomic %) of the individual elements observed on (CH_3_NH_3_)_3_SbBiI_9_/TiO_2_ substrate shown in Fig. 4e

Spot	Antimony	Bismuth	Iodine	Titanium	Gold
Point 1	2.70	3.11	23.86	68.98	1.32
Point 2	11.18	8.04	74.57	3.18	3.00
Point 3	9.09	7.42	65.20	11.73	6.23
Point 4	0.00	3.01	29.55	60.94	6.35
Point 5	0.00	3.76	27.73	65.99	2.50

**Table 6:** Elemental distribution (atomic %) of the individual elements observed on (CH_3_NH_3_)_3_SbBiI_9_/NiO substrate shown in Fig. 4f

Spot	Antimony	Bismuth	Iodine	Nickel	Gold
Point 1	6.36	8.85	74.85	2.55	7.36
Point 2	1.32	8.68	71.86	5.81	11.76
Point 3	0.00	0.00	3.58	38.82	57.58
Point 4	9.54	9.71	75.48	0.12	5.13
Point 5	3.83	9.61	60.83	7.86	17.85

**Fig. 5:**
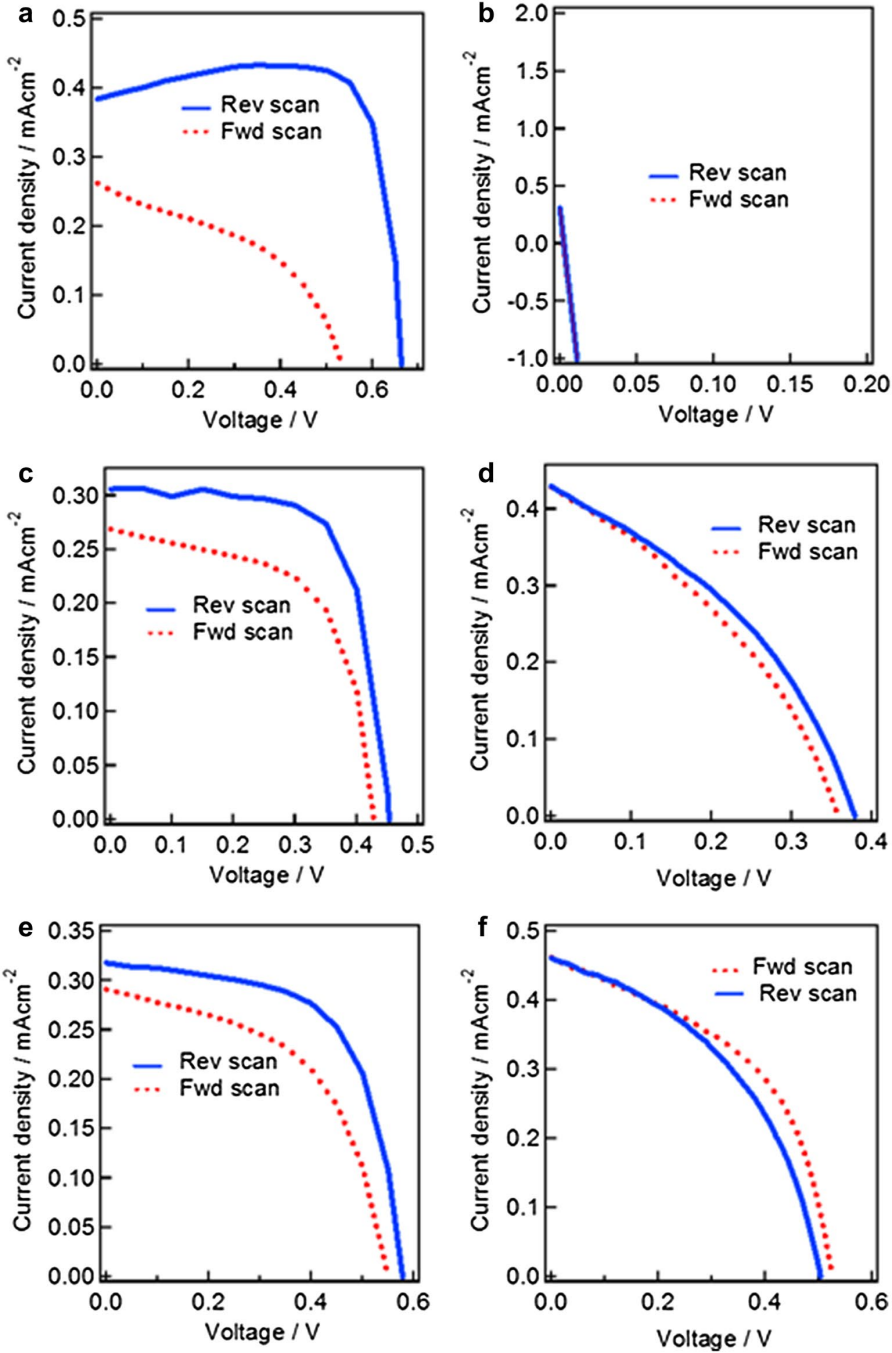
Photo I–V curves of lead-free A_3_B_2_X_9_-crystal solar cells measured under simulated irradiation (AM 1.5, 100 mW cm^−2^) as normal mesoscopic and inverted architectures respectively. The photoabsorption layers were (CH_3_NH_3_)_3_Bi_2_I_9_ (**a**, **b**), (CH_3_NH_3_)_3_Sb_2_I_9_ (**c**, **d**), and (CH_3_NH_3_)_3_SbBiI_9_ (**e**, **f**) crystal films. The photovoltaic characteristics were summarized in Table 7

Figure 5 shows the photo I–V characteristics of solar cells with best PCE using the lead-free photo absorbing materials ((CH_3_NH_3_)_3_Bi_2_I_9_, (CH_3_NH_3_)_3_Sb_2_I_9_, and (CH_3_NH_3_)_3_SbBiI_9_) with different structure (n-i-p and p-i-n, in Fig. 1), measured under the simulated sun light (AM 1.5, 100 mW cm^−2^). The observed PCE parameters are summerized in Table 7 and are significantly lower than the standard perovskite solar cells using CH_3_NH_3_PbI_3_ [1–3], which would be attributed to the uneven surface morphology of lead free perovskite solar cells. Basically, the cells using porous-TiO_2_ electrode gave better *FF*s, which is due to the prohibition of short circuiting by porous TiO_2_ layer [29]. Actually, the cells without A_3_B_2_I_9_ conformal layers on porous TiO_2_ [(CH_3_NH_3_)_3_Sb_2_I_9_ (Table 3) and (CH_3_NH_3_)_3_SbBiI_9_ (Table 5)] also provide better *FF*s. On the other hand, the cells using NiO provide small *FF*s. Specially, the cells using (CH_3_NH_3_)_3_Bi_2_I_9_ on NiO performed the short circuitting (Fig. 5b, Table 2). However, in spite of the missing points of (CH_3_NH_3_)_3_Sb_2_I_9_ and (CH_3_NH_3_)_3_SbBiI_9_ on NiO (Tables 4, 6), the I–V curves in Fig. 5d and f show *FF*s over 0.3. The prohibition of short circuiting would be due to the slight presence of Sb^3+^ on NiO surface, which was not detected by the EDX analysis. It was interesting that the hysteresis of A_3_B_2_X_9_ solar cells on NiO layer were smaller than those on TiO_2_ layer, as CH_3_NH_3_PbI_3_ solar cells [30].

**Table 7:** Photovoltaic performance of (CH_3_NH_3_)_3_Bi_2_I_9_, (CH_3_NH_3_)_3_Sb_2_I_9_, (CH_3_NH_3_)_3_SbBiI_9_ photon harvesting layer based mesoscopic normal and inverted planar architecture solar cells

Photo-layer	Architecture	Scan direction	Efficiency/%	Jsc/mAcm^−2^	Voc/V	FF
(CH_3_NH_3_)_3_Bi_2_I_9_	Normal	Rev	0.21 (0.22)	0.40 (0.38)	0.64 (0.68)	0.81 (0.88)
		Fwd	0.07 (0.06)	0.29 (0.26)	0.53 (0.53)	0.45 (0.43)
(CH_3_NH_3_)_3_Bi_2_I_9_	Inverted	Rev	0.00 (0.00)	0.27 (0.30)	0.00 (0.00)	–
		Fwd	0.00 (0.00)	0.22 (0.25)	0.00 (0.00)	–
(CH_3_NH_3_)_3_Sb_2_I_9_	Normal	Rev	0.080 (0.095)	0.25 (0.30)	0.45 (0.45)	0.70 (0.69)
		Fwd	0.057 (0.07)	0.21 (0.26)	0.43 (0.42)	0.62 (0.61)
(CH_3_NH_3_)_3_Sb_2_I_9_	Inverted	Rev	0.058 (0.061)	0.45 (0.43)	0.38 (0.38)	0.36 (0.37)
		Fwd	0.057 (0.055)	0.45 (0.42)	0.36 (0.35)	0.35 (0.35)
(CH_3_NH_3_)_3_SbBiI_9_	Normal	Rev	0.099 (0.11)	0.27 (0.31)	0.59 (0.57)	0.64 (0.62)
		Fwd	0.067 (0.084)	0.23 (0.29)	0.55 (0.54)	0.55 (0.52)
(CH_3_NH_3_)_3_SbBiI_9_	Inverted	Rev	0.05 (0.10)	0.44 (0.46)	0.32 (0.50)	0.34 (0.44)
		Fwd	0.06 (0.11)	0.44 (0.46)	0.34 (0.52)	0.35 (0.47)

Data shown here represent the average of three independent solar cell parameters. Best representative photovoltaic parameters are shown in the bracket

### A_3_BX_6_ crystals with Ag or Cu cation for the A site

**Fig. 6:**
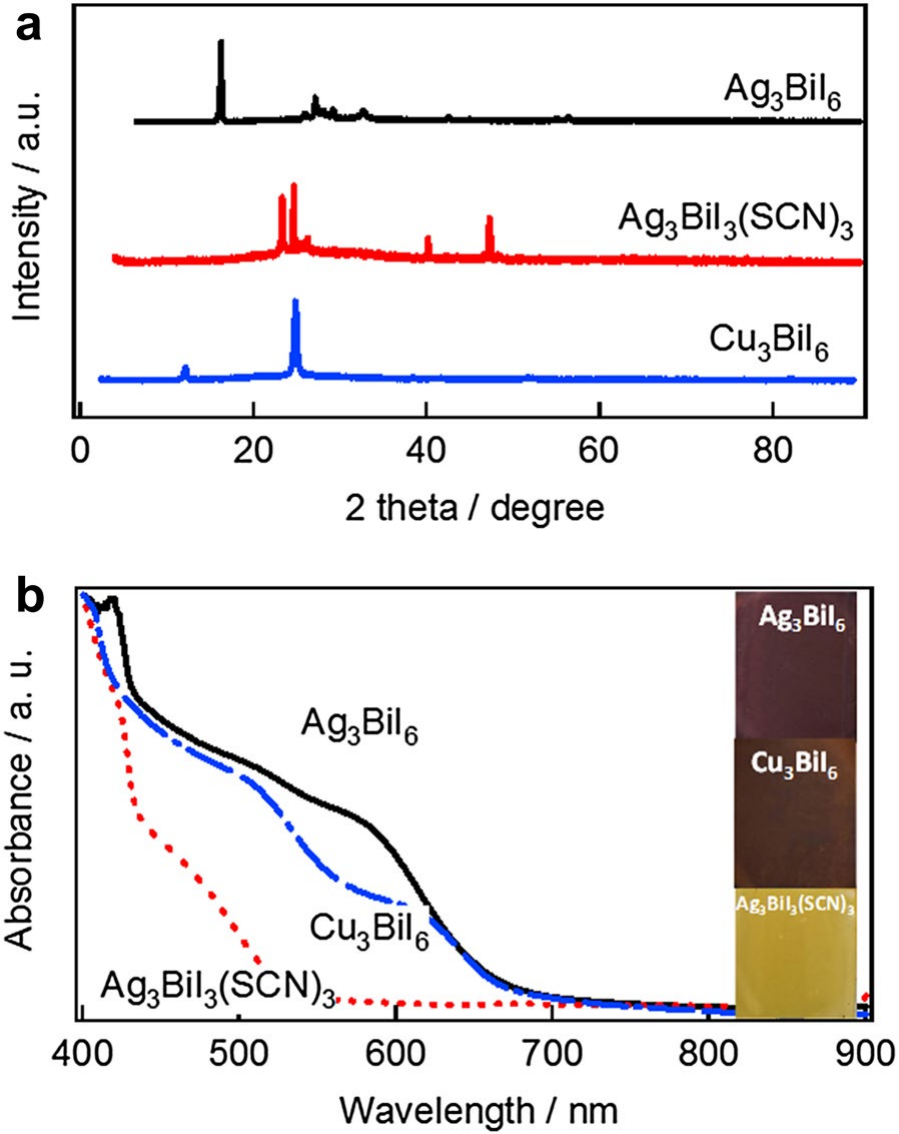
XRD patterns (**a**) and UV–vis absorption spectra (**b**) of Ag_3_BiI_6_, Ag_3_BiI_3_(SCN)_3_ and Cu_3_BiI_6_ casted on the glass substrates. The insets were the pictures of Ag_3_BiI_6_, Ag_3_BiI_3_(SCN)_3_ and Cu_3_BiI_6_ films

Copper and silver are non-toxic material and especially, Cu^+^ is a cheap and earth abundant one. The structure of A_3_BX_6_ was regulated by the ratio of elemental materials in the DMSO solution. Hence, CuI and BiI_3_ were mixed in 3:1 molar ratio to be Cu_3_BiI_6_. AgI and BiI_3_ were mixed in 3:1 molar ratio to be Ag_3_BiI_6_. In case of Ag_3_BiI_3_(SCN)_3_, AgSCN and BiI_3_ were mixed in 3:1 molar ratio. The XRD patterns of Ag_3_BiI_6_, Ag_3_BiI_3_(SCN)_3_ and Cu_3_BiI_6_ as casted films are shown in Fig. 6a. The film made by Ag_3_BiI_6_ signifies the trigonal structure with R $\overline{3}$ m space group [31]. The space group determination of relatively new materials Ag_3_BiI_3_(SCN)_3_ and Cu_3_BiI_6_ could not be realized. Now, we are analyzing the exact crystal structures of Ag_3_BiI_3_(SCN)_3_ and Cu_3_BiI_6_ as the further research works. Figure 6b shows the UV–vis absorption spectra observed. The Ag_3_BiI_6_ and Cu_3_BiI_6_ films show higher absorption than the Ag_3_BiI_3_(SCN)_3_ one. Due to the absorbance onset of Ag_3_BiI_6_ and Cu_3_BiI_6_ at around 680 nm, the films were dark brown. On the other hand, Ag_3_BiI_3_(SCN)_3_ film was pale yellowish. The difference of Ag_3_BiI_6_ and Cu_3_BiI_6_ were the variation of spectra at around 580 nm. The exact absorption coefficients were not shown in the figure due to the inhomogeneous structures of films, which will be shown later.

**Fig. 7:**
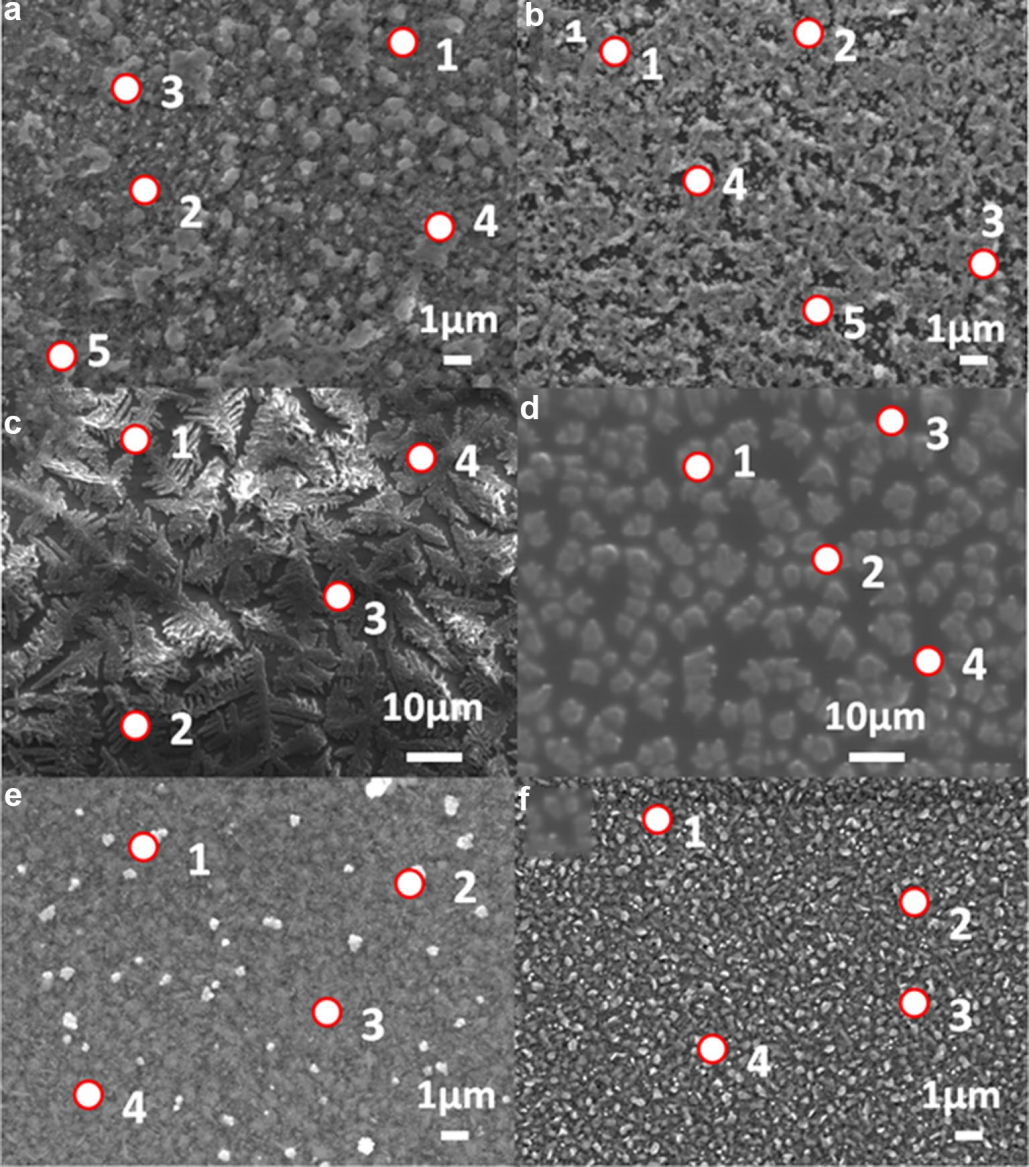
SEM images observed for elemental analysis of A_3_BX_6_ films [Ag_3_BiI_6_: (**a**, **b**); Ag_3_BiI_3_(SCN)_3_: (**c**, **d**); Cu_3_BiI_6_: (**e**, **f**)] grown on different substrates [TiO_2_: (**a**, **c**, **e**); NiO: (**b**, **d**, **f**)]. The marked spots indicate the positions of elemental analysis, which is related to the results shown in Tables 8, 9, 10, 11, 12 and 13

Figure 7 shows the surface morphologies of Ag_3_BiI_6_, Ag_3_BiI_3_(SCN)_3_ and Cu_3_BiI_6_ films on porous-TiO_2_ and planer-NiO substrates which were observed by the SEM. Although the Ag_3_BiI_6_ films seems to be uniform observed by eyes, the nanoscale morphology in this SEM image scaled at 1 µm was relatively uniform (Fig. 7a). It was noticed that there were 1 μm-sized small grains of Ag_3_BiI_6_ on porous-TiO_2_ (Fig. 7a). However, Ag_3_BiI_6_ on planer NiO became the flakey layer with cracks, and exact crystals were not observed in the SEM image (Fig. 7b). For the other silver cation and mixed anion based Ag_3_BiI_3_(SCN)_3_ film, the SEM image with 10 μm scale shows the leaf-like crystal pattern on porous-TiO_2_ substrate and the grain structure on planer-NiO substrate with space between the crystals as the non-uniform substrate coverage resulting in the limitation in photon harvesting and charge collection (Fig. 7c, d). The morphologies of Cu_3_BiI_6_ were small grains with around 0.1–0.5 μm which were dispersed homogeneously on porous-TiO_2_ and planer-NiO substrates (Fig. 7e, f).

The individual elemental distribution (EDX analysis) involved of surface morphology of Ag_3_BiI_6_, Ag_3_BiI_3_(SCN)_3_ and Cu_3_BiI_6_ films are shown in Tables 8, 9, 10, 11, 12 and 13. The analyses spots were chosen randomly and are shown on its SEM images in Fig. 7. Although A_3_BX_6_ crystals of Ag_3_BiI_6_, Ag_3_BiI_3_(SCN)_3_ and Cu_3_BiI_6_ distributed relatively homogeneously than A_3_B_2_X_9_ ones using CH_3_NH_3_^+^ cations (Fig. 3 and Tables 1, 2, 3, 4, 5, 6), still the materials were dispersed with inhomogeneity about the elemental ratio (Tables 8, 9, 10, 11, 12, 13). It can be noticed that the amount of Bi was smaller than A site monovalent cations (Ag^+^ and Cu^+^). Specially, it was prominent that the amount of Ag^+^ was quite higher than that of Bi^3+^, which should be improved for the further progress.

**Table 8:** Elemental distribution (atomic %) of the individual elements observed on a particular spot on Ag_3_BiI_6_/TiO_2_ substrate shown in Fig. 8a

Spot	Bismuth	Silver	Iodine	Titanium	Oxygen	Silicon
Point 1	4.75	32.89	33.54	11.97	9.11	7.75
Point 2	2.01	65.52	16.29	7.19	5.76	3.24
Point 3	2.73	20.47	19.50	15.86	19.65	21.79
Point 4	1.80	53.99	18.38	10.09	11.56	4.18
Point 5	5.03	43.57	26.93	15.62	3.98	4.87

**Table 9:** Elemental distribution (atomic %) of the individual elements observed on a particular spot on Ag_3_BiI_6_/NiO substrate shown in Fig. 8b

Spot	Bismuth	Silver	Iodine	Nickel	Oxygen	Silicon
Point 1	2.00	32.49	10.34	1.06	14.54	39.57
Point 2	4.88	40.11	20.99	1.04	6.98	25.99
Point 3	2.87	45.38	23.67	1.10	6.98	19.99
Point 4	0.83	29.68	5.36	0.69	24.20	39.25
Point 5	0.58	89.08	4.83	0.30	3.55	1.65

**Table 10:** Elemental distribution (atomic %) of the individual elements observed on Ag_3_BiI_3_(SCN)_3_/TiO_2_ substrate shown in Fig. 8c

Spot	Bismuth	Silver	Iodine	Sulphur	Carbon	Nitrogen	Titanium	Oxygen	Silicon
Point 1	3.10	20.88	10.24	2.54	7.94	3.40	12.17	19.97	21.76
Point 2	3.31	45.94	15.54	2.72	6.08	–	11.52	8.57	6.33
Point 3	3.71	29.29	12.62	2.97	8.06	0.84	13.16	12.64	16.71
Point 4	3.98	26.85	8.77	3.29	7.04	4.89	13.25	18.52	13.40

**Table 11:** Elemental distribution (atomic %) of the individual elements observed on Ag_3_BiI_3_(SCN)_3_/NiO substrate shown in Fig. 8d

Spot	Bismuth	Silver	Iodine	Sulfur	Carbon	Nitrogen	Nickel	Oxygen	Silicon
Point 1	3.23	7.19	13.82	5.35	19.35	4.71	0.48	25.38	20.50
Point 2	2.40	41.86	13.76	2.61	8.85	–	0.65	11.82	18.04
Point 3	0.58	16.57	0.91	0.71	10.50	–	0.79	42.75	27.19
Point 4	0.60	16.75	0.84	0.78	11.19	–	0.85	39.73	19.26

**Table 12:** Elemental distribution (atomic %) of the individual elements observed on Cu_3_BiI_6_/TiO_2_ substrate shown in Fig. 8e

Spot	Copper	Bismuth	Iodine	Titanium	Oxygen	Silicon
Point 1	16.38	1.59	22.26	8.54	32.17	19.06
Point 2	9.18	1.65	17.42	9.76	37.88	24.11
Point 3	6.21	1.48	13.18	8.04	42.66	28.44
Point 4	3.20	1.90	9.40	8.35	47.70	29.45

**Table 13:** Elemental distribution (atomic %) of the individual elements observed on Cu_3_BiI_6_/NiO substrate shown in Fig. 8f

Spot	Copper	Bismuth	Iodine	Nickel	Oxygen	Silicon
Point 1	2.19	0.46	3.75	0.57	56.42	36.61
Point 2	4.18	1.03	12.15	0.71	48.00	33.92
Point 3	1.94	0.48	3.77	0.66	51.91	41.23
Point 4	3.29	1.84	7.70	0.52	52.41	34.24

**Fig. 8:**
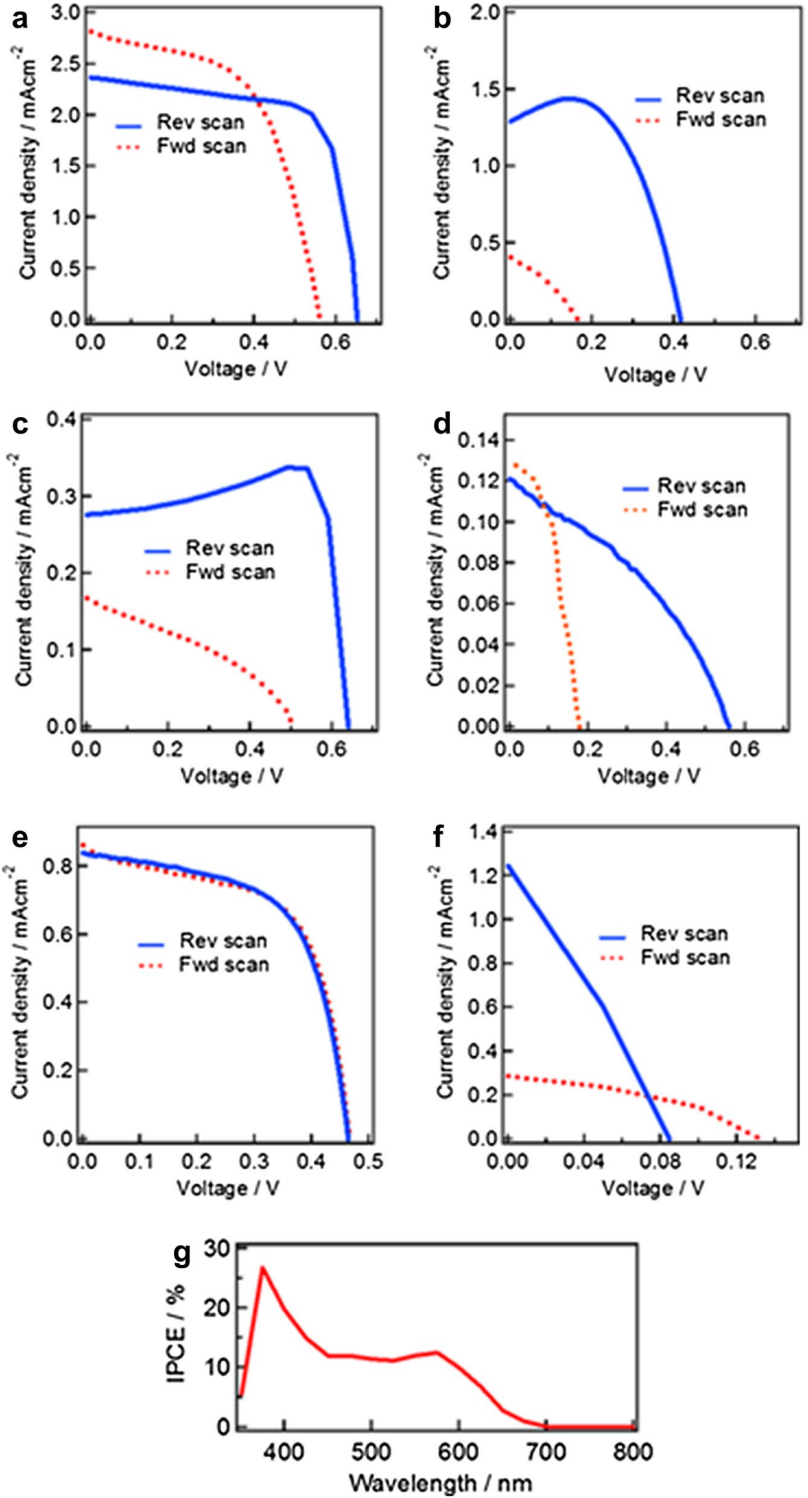
Photo I–V curves of lead-free A_3_BX_6_-crystal solar cells measured under simulated irradiation (AM 1.5, 100 mW cm^−2^) as normal mesoscopic and inverted architectures respectively. The photoabsorption layers are Ag_3_BiI_6_ (**a**, **b**), Ag_3_BiI_3_(SCN)_3_ (**c**, **d**), and Cu_3_BiI_6_ (**e**, **f**) crystal films, and IPCE spectrum of the mesoscopic structure with the Ag_3_BiI_6_ layer (**g**). The photovoltaic characteristics were summarized in Table 14

The photo I–V characteristics of these A_3_BX_6_ (Ag_3_BiI_6_, Ag_3_BiI_3_(SCN)_3_ and Cu_3_BiI_6_) solar cells with different structure (n-i-p and p-i-n, in Fig. 1) under simulated irradiation of 1 SUN are shown in Fig. 8, which were the I–V curves for the best PCE in the series. The photovoltaic parameters were summarized in Table 14. Basically, *V*oc using porous-TiO_2_ layer were higher than using planer-NiO one. The effect of hysteresis on NiO layer was larger, which were different from A_3_B_2_X_9_ [(CH_3_NH_3_)_3_Bi_2_I_9_, (CH_3_NH_3_)_3_Sb_2_I_9_, and (CH_3_NH_3_)_3_SbBiI_9_] solar cells (Fig. 5). But, specially, the cells using Ag_3_BiI_6_ on NiO and Ag_3_BiI_3_(SCN)_3_ on TiO_2_ perform the strong hysteresis and large overshooting at the reverse voltage scanning, which is the overestimation of photovoltaic results. The best *J*_SC_ and PCE in this work using trivalent B-site cation crystals are shown in Fig. 8a with the cell configuration of <FTO/cp-TiO_2_/mp-TiO_2_/Ag_3_BiI_6_/spiro-OMeTAD/Au>. Although there was the hysteresis, the variation was not large as other combinations. The observed PCE (reverse scan) of Ag_3_BiI_6_ based photon harvesting layer attained 1.08% (Fig. 8a, Table 14), which is comparable of reported PCE using AgBi_2_I_7_ based photo absorber [32]. This relatively-high PCE would be due to the pinhole-less surface morphology. However, the association of Ag_3_BiI_6_ layer in inverted architecture could result in only 0.32% PCE (Fig. 8b and Table 14). The IPCE of the Ag_3_BiI_6_ based mesoscopic solar cell is shown in Fig. 8g, the close matching of Jsc of 1.78 mA cm^−2^ is observed. We tried to measure the IPCE of other lead free solar cell, but IPCE measurements could not be managed due to the low *J*_SC_ values. It was interesting that there was negligible hysteresis at the photo I–V measurements of <FTO/cp-TiO_2_/mp-TiO_2_/Cu_3_BiI_6_/spiro-OMeTAD/Au> cells, which would have the significance for the further research efforts in the future.

**Table 14:** Photovoltaic performance of Ag_3_BiI_6_, Ag_3_BiI_3_(SCN)_3_ photon harvesting layer based mesoscopic normal and inverted planar architecture solar cells (Fig. 8g)

Photo-layer	Architecture	Scan direction	Efficiency/%	Jsc/mAcm^−2^	Voc/V	FF
Ag_3_BiI_6_	Normal	Rev	0.91 (1.08)	1.92 (2.36)	0.63 (0.65)	0.75 (0.70)
		Fwd	0.58 (0.88)	1.92 (2.81)	0.52 (0.56)	0.56 (0.55)
Ag_3_BiI_6_	Inverted	Rev	0.14 (0.32)	0.99 (1.29)	0.25 (0.41)	0.42 (0.59)
		Fwd	0.02 (0.02)	0.39 (0.40)	0.14 (0.16)	0.31 (0.32)
Ag_3_BiI_3_(SCN)_3_	Normal	Rev	0.14 (0.18)	0.32 (0.27)	0.63 (0.63)	0.72 (1.00)
		Fwd	0.02 (0.03)	0.21 (0.16)	0.40 (0.50)	0.30 (0.35)
Ag_3_BiI_3_(SCN)_3_	Inverted	Rev	0.007 (0.02)	0.12 (0.12)	0.17 (0.55)	0.34 (0.36)
		Fwd	0.02 (0.01)	0.11 (0.13)	0.46 (0.17)	0.42 (0.46)
Cu_3_BiI_6_	Normal	Rev	0.19 (0.23)	0.69 (0.83)	0.45 (0.46)	0.59 (0.60)
		Fwd	0.19 (0.23)	0.71 (0.86)	0.46 (0.46)	0.57 (0.59)
Cu_3_BiI_6_	Inverted	Rev	0.028 (0.03)	1.70 (1.24)	0.06 (0.08)	0.27 (0.29)
		Fwd	0.016 (0.017)	0.32 (0.28)	0.13 (0.13)	0.39 (0.45)

Data shown here represent the average of three independent solar cell parameters. Best representative photovoltaic parameters are shown in the bracket

## Conclusions

In summary, we fabricated the lead-free hybrid solar cells in standard mesoscopic and inverted architecture incorporating various solution processed photon harvesting layer of (CH_3_NH_3_)_3_Bi_2_I_9_, (CH_3_NH_3_)_3_Sb_2_I_9_, (CH_3_NH_3_)_3_SbBiI_9_, Ag_3_BiI_6_, Ag_3_BiI_3_(SCN)_3_ and Cu_3_BiI_6_. The best PCE results was obtained using <FTO/cp-TiO_2_/mp-TiO_2_/Ag_3_BiI_6_/spiro-OMeTAD/Au> structure with 1.08% of power conversion efficiency (PCE). The grown morphology of A_3_B_2_X_9_ and A_3_BX_6_ crystals can be different on the substrates (porous TiO_2_ or planar NiO layers). The unevenness can be the hindrance for the improvement of photovoltaic effects. Hence, it is advisable to use lead-free element which can show uniform coverage of the substrate.

Other interesting findings in this report were the prohibition of short circuiting of solar cells using Sb deposition on NiO layer and the diminishment of photo I–V hysteresis using porous-TiO_2_/Cu_3_BiI_6_ combination, which can be considered for the further progresses.

## Experimental details

All chemicals were of reagent grade quality and used without any further processing. Antimony iodide (SbI_3_) was purchased from Yanagishima Pharmaceutical Co. Ltd. Bismuth iodide (BiI_3_), Silver iodide (AgI), Nickel (II) acetylacetonate, Phenyl-C61-butyric acid methyl ester (PCBM) and bathocuproine (BCP) were purchased from Aldrich. Copper(I) iodide (CuI) from Kanto Chemical Ltd., Silver thiocyanate (AgSCN) from Wako Pure Chemical Industries Ltd., and Methylammonium iodide was purchased from TCI respectively. Dimethyl sulfoxide (DMSO), Chlorobenzene, and Methanol were purchased from Wako. γ-Butyrolactone (GBL) and Acetonitrile were procured from Chameleon Reagent and Kanto Chemical Co. Inc. respectively.

FTO glass (TEC-15, t = 2.1 mm), purchased from Pilkington were cut in an appropriate size and cleaned ultrasonically with detergent water, distilled water and ethanol, respectively each for 15 min. The cleaned FTO glass was passed through UV-O_3_ treatment for 10 min to remove the organic impurities. To fabricate the standard mesoscopic n-i-p solar cell, diluted TAA solution (Titanium di isopropoxide bis(acetylacetonate)/Sigma-Aldrich) of (200 µL in 7.5 mL ethanol) was aerosol spray coated maintaining the substrate temperature 500 °C. After arriving the ambient temperature the coated FTO glass was passed through the UV-O_3_ treatment and, diluted PST-30NRD TiO_2_ solution (1:3.5 wt/wt) was coated by spinning process at 5000 rpm for 30 s. The mesoporous TiO_2_ coated substrate was dried out at 120 °C for 5 min and again baked at 500 °C for 30 min in a furnace.

To make the perovskite layer, the substrate was treated for 5 min for UV-O_3_ treatment and transferred inside N_2_ filled glove box. To prepare the (CH_3_NH_3_)_3_Sb_2_I_9_ film, SbI_3_ and methylammonium iodide was mixed in DMSO:GBL (1:1) solvent and 0.25 M concentration of (CH_3_NH_3_)_3_Sb_2_I_9_ was maintained by keeping the molar ratio as CH_3_NH_3_I:SbI_3_ = 3:2 molar ratio. To prepare the (CH_3_NH_3_)_3_Bi_2_I_9_ film, BiI_3_ and methylammonium iodide was mixed to maintain the 0.25 M concentration of (CH_3_NH_3_)_3_Bi_2_I_9_ in DMSO:GBL (1:1) by keeping the molar ratio as CH_3_NH_3_I:BiI_3_ = 3:2. To prepare the (CH_3_NH_3_)_3_SbBiI_9_ film, SbI_3_, BiI_3_ and methylammonium iodide was mixed to maintain the 0.25 M concentration of (CH_3_NH_3_)_3_SbBiI_9_ in DMSO:GBL (1:1) by keeping the molar ratio as CH_3_NH_3_I:SbI_3_:BiI_3_ = 3:1:1. Semiconductor Ag_3_BiI_6_ layer overcoated TiO_2_ mp substrate was prepared by making a 0.5 M solution of AgI and BiI_3_ in DMSO solvent with 0.5 M concentration of Ag_3_BiI_6_ by keeping the molar ratio as AgI:BiI_3_ = 3:1. To fabricate and explore the photon harvesting properties of Ag_3_BiI_3_(SCN)_3_, BiI_3_ and Silver thiocyanate (AgSCN) was mixed in 1:3 molar ratio to maintain the 0.5 M concentration in DMSO solvent. Copper based photon harvesting layer (Cu_3_BiI_6_) was prepared by mixing the CuI and BiI_3_ in DMSO solvent with 0.4 M concentration of Cu_3_BiI_6_ by keeping the molar ratio as CuI:BiI_3_ = 3:1.

In all cases, the solution temperature was maintained at 80 °C and the heated solution was spun on mp TiO_2_ substrate with 2000 rpm for 30 s and preserved on a hot plate at 80 °C for 30 min to grow the respective photo absorber layer.

To collect the holes smoothly a hole selective layer spiro-OMeTAD (2,2,7,7-Tetrakis(*N*,*N*di-p-methoxy phenylamine)-9,9-spirobifluorene)of 28.9 mg in 400 µL chlorobenzene, with additives [11.5 µL t-butyl pyridine (Sigma-Aldrich), 7 µL Li-TFSI (520 mg in 1 mL acetonitrile) and 8.8 µL Co Complex (40 mg in 0.1 mL acetonitrile)] was spun on a cold substrate of lead-free prepared semiconducting layer at 4000 rpm for 20 s and stored for drying under dark. Finally, gold layer was thermally evaporated and deposited to maintain 80 nm thickness, which works as a metal back contact layer.

The inverted structure hybrid solar cell was fabricated by coating Nickel (II) acetylacetonate in acetonitrile (0.04 M) solution by spray process while maintaining the UV-O_3_ treated FTO glass temperature at 500 °C. After reaching the ambient temperature the coated NiO substrate was transferred to N_2_ filled glove box for lead-free semiconductor layer preparation. The preparation of individual photo harvesting layer was followed as described previously. On top of the lead-free photo absorber layer, a 20 mg/mL PCBM in chlorobenzene was spun at 1000 rpm for 60 s and kept under ambient N_2_ for 30 min to dry it out. Subsequently, a BCP solution of 1 mg/mL in methanol was spun at 1000 rpm for 60 s. Finally, a silver metal contact was prepared by thermally evaporation process maintaining its thickness 80 nm.

The I–V characteristics were measured by an AM 1.5G solar simulator equipped with a 500 W Xe lamp (YSS-80A, Yamashita Denso) by placing a black 0.09 cm^2^ mask area. A reference Si photodiode (Bunkou keiki co. ltd., Japan) was used to calibrate the power of the solar simulator light. The I–V characteristics were obtained by applying an external bias to the fabricated solar cell and measurement of generated photocurrent was performed with a DC voltage current source (Agilent, B2901A). SEM images were obtained by HITACHI Microscope TE3030 and EDX analysis was performed with Oxford Instruments, X-stream-2.
